# Non-operative management of splenic trauma

**Published:** 2012-03-05

**Authors:** M Beuran, I Gheju, MD Venter, RC Marian, R Smarandache

**Affiliations:** “Carol Davila” University of Medicine and Pharmacy, Bucharest, Romania; 3rd Department of Surgery, Clinical Emergency Hospital, Bucharest, Romania

**Keywords:** spleen, blunt trauma, conservative approach

## Abstract

The risk of overwhelming postsplenectomy infection (OPSI) prompted the evolution toward preservation of the injured spleen. Nonoperative management (NOM) of blunt injury to the spleen in adults has become the standard of care in hemodynamically stable patients. This modality of treatment began in the 1970’s in paediatric patients. It is highly successful with overall failures rates from 2% to 31% (average 10.8%) - with the majority of failures occurring in the first 24 hours. Current, NOM of splenic trauma includes splenic artery embolization.

However, the criteria for NOM are controversial. In this study we present the current criteria, the evolution and failure rates of this type of management viewed through the general knowledge and, particularly, our experience.

Billroth suggested over 100 years ago that the injured spleen has the ability of self-healing [**1**]. He submitted this theory following the post-mortem findings in a 43 year-old woman, who fell from height during work and died 5 days later from brain and abdominal injuries. The autopsy revealed a splenic injury without an obvious sign of recent bleeding. Therefore, Billroth concluded that ”the lesion healed completely judging by the macroscopic appearance of the lesion and the reduced amount of intraperitoneal blood”.

When it comes to visceral injuries following abdominal trauma, there is nothing as radical as the nonoperative management (NOM) of hepatic and splenic injuries [**2**]. The treatment for blunt abdominal trauma has significantly changed thanks to new diagnostic methods and the accurate assessment of organ damage. 

In order for nonoperative treatment (NOT) of splenic injuries to be the standard goal of therapy in hemodinamically stable patients, it is necessary to have an accurate knowledge of patient selection criteria for nonoperative management, as well as a precise assessment of the factors precluding conservative therapy. This becomes tangible due to diagnostic and therapeutic angiography addition.

This major therapeutic change was the consequence of many clinical studies indicating that splenectomy increases the risk of infection susceptibility with its most deadly manifestation-OPSI (overwhelming postsplenectomy sepsis), which appears in 0.5% of all splenectomies in trauma patients and in over 20% of elective splenectomies for hematologic disorders [**3**]. OPSI is most frequent during the first 2 years of asplenia but there is a permanent risk of infection with a mortality of over 80%.

Knowing all these factors set the trend in splenectomy-conservative therapy debate (non-operative management, conservative surgery, and spleen auto transplant); it is currently considered that traumatic splenic injury is no longer an absolute indication for splenectomy, thus a proper reviewing of indications for emergency surgery in traumatic hemoperitoneum is needed.

In 1882, Gross indicated NOM for spleen injuries and recommends bed rest and mild diet for minor injuries and lead acetate, ergot and opium for severe lesions; surgery will be performed only if necessary. “A System of Surgery” [**4**].

According to Lucas [**1**] the pioneer for non-operative management (NOM) in splenic trauma was Wanborough, in 1940 (Sick Children's Hospital Toronto).

In 1968, Upadhyaya observed that “often, children with splenic injuries do not exhibit the signs of an important blood loss. It is intriguing that in most children with splenic lesions the bleeding had stopped by the time of laparotomy “ [**4**]. This fact is explained by varied mechanisms: hypotension, clot formation, the gluing effect of the great omentum, perisplenic hematoma containing the actual bleeding, intact splenic capsule.

In 1971, Douglas and Simpson (Toronto Hospital for Sick Children) described 32 cases of children with clinical signs of splenic injury treated conservatively out of whom, 25 children did not require surgical intervention. This study proved that the spleen has indeed the capability of healing itself with an excellent outcome in selected cases (Douglas cit.5).

However, employing non-operative treatment for splenic injuries in adults was initially a challenge for surgeons for several reasons: the post-splenectomy sepsis is less frequent and less severe compared to children; structural and vascular splenic changes according to age and possibly the type of force inducing the lesion make a spontaneous hemostatis unlikely; the risk of overlooked associated injuries; the ensuing possibility of delayed rupture of the spleen (DRS), splenosis or post-traumatic cyst [**6**].

Other explications, although not scientifically founded, include a much thinner and somewhat less elastic splenic capsule in adults (Morgenstern,Gross cit. 7), lesion disposition in relation to splenic vasculature (much more favorable when the lesion is parallel with the blood vessels), associated rib fractures. These discrepancies are explained by an increased severity of adult trauma which usually associates extra and intra-abdominal injuries requiring surgical intervention (1997 Powell cit. 8).

Patients with traumatic splenic injuries can be treated surgically or conservatively according to the surgeon’s or hospital’s characteristics. Britt [**9**] first uses the term of “alternative surgery” to define non-operative management or the selective approach of trauma patients.

NOM represents the progression of S.O.S. concept (save our spleens), which was initially used for children and later on extended to adults.

Patients with traumatic spleen injuries have a significantly higher risk of bleeding than those with hepatic injuries (5% vs 1% -10).

Eastern Association for Surgery of Trauma (EAST) infers that “non-operative management of splenic and/or hepatic trauma in hemodinamically stable patients is feasible” [**10**]. 54.8% of the patients included in this study have been successfully treated conservatively; there were 10.8% failed approaches with 60.9% of them materializing in the first 24 hrs.

Splenic conservation after blunt abdominal trauma became possible thanks to the following findings:

• Whitessell observed that splenic fractures following blunt abdominal trauma are most frequently perpendicular on the organ’s long axis, therefore the risk of segmental vascular damage is quite small (the intersegmental avascular planes) (cit. 7);

• Upadhyaya and Simpson observed that transverse splenic fractures in children do not exhibit active bleeding when splenectomy was performed, suggesting that hemostatic surgical intervention was not necessary;

o The medical community acknowledged the important immunological role of the spleen;

o Improvement of non-invasive diagnostic methods (especially CT).

When employing NOM it is necessary to select the patients with hemorrhagic lesions that clot spontaneously (longitudinal lesions that parallel the long axis of the spleen may cross larger segmental vessels with an unlikely possibility of spontaneous hemostasis).

The standard criteria for NOM are [**11,12**]:

• hemodynamic stability/ readily stabilizable;

• lack of rebound and guarding;

• blood transfusions ≤ 4 units;

• no lack of consciousness;

• age < 55 years;

• imagistically documented splenic injury.

The only absolute indication for emergency laparotomy is hemodynamic instability [**11,12**].

Complex/severe splenic injuries, age, pre-existent splenic diseases, number of units of transfused blood, brain injuries are no longer considered absolute contraindications for NOM (13,14,Gaunt,Avanoglou-cit.11,15,16,17).

“NOM for blunt splenic injuries replaces splenorrhaphy which was the usual method for preserving the spleen” [**13**]; Garber [**18**] is the author of a multicentric retrospective study, made in Ontario (Canada) which validates that NOM is the preferred therapeutic method (in 69% of patients), followed by splenectomy (28%) and splenorrhaphy (4%) in non-trauma centers and 65%, 33% and 2% respectively in trauma centers. The incidence of NOM has increased from 59% (1991) to 75% (1994) and that of splenectomy has decreased from 35% (1991) to 24% (19914). The incidence of splenorrhaphy has significantly dropped from 6% to 1%.

Even 2 units of transfused blood during the first 48 hrs (in order to maintain a HGB level above 8 g/dl) is compatible with a successful NOM [**1,3**].

In penetrating anterior abdominal injuries (with splenic injury) NOM is applicable only if the patient presents:

• hemodynamic stability;

• lack of rebound and guarding;

• no evisceration;

• no orifice/multiple orifice bleeding [**9**].

It was initially considered that patients undergoing NOM or splenorrhaphy require bed rest for 1 week and avoidance of physical activity for 6 months; the experimental studies performed on dogs and pigs by Dulchavsky and co. showed that splenic scarring consists of an extensive capsular fibrosis and fibrous reaction at splenorrhaphy site and paralleling intrasplenic septs [**19**]. Kluger [**20**] performed an experimental study on young rats and adult rats in order to clear up the cellular mechanism of splenic scarring after trauma and the influence of patient’s age on the success of NOM.

He observed that the local bleeding reabsorbed in the first 48 hrs in young rats and in 7 days in adult rats; he also noticed that splenic parenchyma regeneration appeared in 14 days in young rats whilst in adult ones the process was incomplete by the 21st day. Peak accumulation of myofibroblasts at the laceration site took place during day 2 in young rats and during day 4 in adult ones. Splenic lacerations heal through a regeneration process and not by collagen scarring.

Accelerated splenic healing that grants a successful NOM in children and young adults is explained by this early accumulation of myofibroblasts at the lesion site. Benya [**21**] conducted a study that included children with grade I-II splenic injuries with complete resolution on CT scans after 4 months from the initial injury; for severe lesions the healing time is extended to over 6 months for grade III and over 11 months for grade IV injuries.

The author considers a complete resolution on CT scan when there are no abnormal areas in or around the spleen or when there is a mild residual deformation of the splenic outline (without the obvious presence of a hematoma/ perisplenic fluid collection).

Patients of at least 50 years of age do not represent a contraindication for NOM, although they are at risk for an unsuccessfull conservative approach [**22**].

According to Longo, Uranüs and Sartorelli [**3,22,23**] predictive parameters for a successful NOM include:

• hemodinamically stable/ readily stabilizable;

• blood transfusions < 4 units;

• age < 55 years;

• early resolution of splenic abnormalities obvious on imagistic investigations;

• no lack of consciousness/ no brain injuries;

• no associated intra- or retroperitoneal injuries (upon abdominal CT scan) that would require surgical intervention;

• no rebound or guarding;

• complete recovery of bowel movements.

Knudson (cit 14) considers that the hemoperitoneum secondary to spleen/ liver injuries is absorbed after the 5th day from the initial insult. If free intraperitoneal blood is still present after day 5 upon CT scan there is the possibility of overlooked injuries or rebleeding.

## Associated extra-abdominal injuries

Blunt aortic injuries accompany hepatic and splenic lesions in 15-20% of cases (Fabian, Hunt cit. 24); Santaniello’s study [**24**] states that 33% of the patients with blunt aortic injury have associated simultaneous hepatic/ splenic lesions. Recent NOM protocols for splenic injuries debunk the “removal of spleen from the equation” myth. Santaniello’s study shows that minor splenic injuries (grade I-II) associated with aortic lesions pose a minimum/no risk for anticoagulation therapy. In this article’s editorial Kenneth Mattox disagrees upon unrecognized these findings when dealing with aortic injury associated with major splenic lesions.

Sartorelli [**23**] considers that the outcome of NOM in multiple parenchymal trauma patients is not different from that of NOM in unique organ involvement. Furthermore, NOM in patients with associated brain injuries to hepatic/ splenic lesions is safe (Archer cit. 23,25). Garber [**18**] observed that chest injuries account for most of the associated lesions (77%), followed by head injuries (59%).

An age over 55 years was considered a criterion for an unsuccessful NOM (Godley had a rate of success of 9% when employing NOT in elderly patients; Esposito cit 23). Why? Elderly patients have diminishing biological reserves; structural alterations concordant with age make a spontaneous hemostasis unlikely, increased splenic frailty. In an attempt to decipher these statements, Barone [**17**] quotes 2 articles written by Morgenstern and published between 1983 and 1979. Morgenstern and Uyeda (1983) assert that “splenic hemostatis is tempered by age, children and young adults having functional smooth muscle tunic and elastic tunic” whilst elderly patients exhibit structural changes that “restrict the contraction and retraction of damaged blood vessels within the splenic parenchyma”.

In 1979, Morgenstern and Shapiro suggested that splenorrhaphy should be contraindicated in elderly patients. In 1964, Gross observed the structural distinction between the splenic capsule in young adults and elderly patients, stating that “after the age of 60 years the splenic capsule is thickening”. Perhaps Gross’s studies should be reviewed and set as a standard protocol for NOM in elderly patients. (Barone-17). Sartorelli [**23**] reported favorable results for NOM in 83.3% of all patients >55 years old, similar to those conveyed by Barone (83%- 17), Myers [**26**], Brasel (71% -15) and Cocanour [**12**]. Furthermore, Clancy [**27**] declared that the percentage of conserved spleens in patients over 65 years of age is similar to that of younger patients (40 patients over 65 years of age have been treated successfully by NOM). It is not the age but the grade of splenic injury that increases the risk of failure for NOM [**28**]. The use of BOAST (Bedside Organ Assessment with Sonography for Trauma) as well as permanent and careful monitoring of these patients ensures the success of favorable outcome with NOM [**29**].

According to Frizis, [**30**] old age has no influence on the final outcome of elderly multiple trauma patients; trauma is not only a disease of the young and “age is the problem, not the injury”. 

**The level of consciousness** - in the past, patients with altered mental status were not treated conservatively because of overlooked intra-abdominal injuries that might require laparotomy. However, Archer’s [**31**] and Keller’s [**25**] juvenile studies did not warrant the existence of undiagnosed complications in children. Rozycki’s study [**29**] corroborates Archer’s findings, including for patients with a GCS ≤ 8, stating that ”NOM is not only perfectly feasible in patients with severe brain damage, but efficient and safe”. According to Pal [**32**] the CT scans represent a very effective diagnostic method for hemodinamically stable patients with altered mental status and equivocal abdominal exam, having a sensitivity of 97.7%, a specificity of 98.5% and an overall accuracy of 99.4%. Authors consider that DPL is not necessary in this group of patients. 

Archer’s results (NOM in patients with altered mental status is safe in a strictly monitored environment) are confirmed by the rate of success of NOM in patients with GCS<13 (93%). Likewise, Cocanour [**12**] considers that brain injuries are not a contraindication for NOM.

Sartorelli’s study [**23**] about multiple intraabdominal parenchymal injuries established a rate of success for NOM of 94.1%, therefore confirming its safety; similarly, Goan [**14**] considers that NOM in patients with hepatic lesions is secure when careful clinical and imagistic monitoring is provided. 

**The severity of splenic injury**- it appears that NOM is effective in splenic injuries with an average lesional AAST score of 3 [**33**]. There are a few studies [**34**] (Nallathambi, Malangoni, Pickhardt, Brick, Mahon, Taylor, Jeffrey cit.34,35) signaling the fact that splenic injuries have an unpredictable progress and proving there is no obvious correlation between the anatomical lesion severity and clinical outcome. Velmahos debated these results based on his conclusions: AIS is a flawed system of staging intra-abdominal visceral injuries; a useful prediction model should be simple.

**The severity of hemoperitoneum**- it is considered to be correlated with the injury score; Hiatt and Federico (cit. 14) considered the exact opposite to be true.

**Blood transfusions > 4 units;** all patients included in Sartorelli’s study with early failure of NOM required more than 4 units of transfused blood. A hemoglobin level of < 9 g/dl and a heart rate of > 100 beats per minute is an indicator for blood transfusion.

Recent protocols for NOM are applicable in all multiple trauma patients with splenic injuries (but without hemorrhage), requiring more than 4 units of transfused units (usually following pelvic fractures) only in trauma centers. It is important to remember that prolonged bleeding may cause clotting disturbances, affecting the overall outcome of NOM, thus emphasizing the importance of an accurate clinical assessment.

Multiple transfusions are actually the hallmark of failed NOM.

Guth and Patcher [**36**] consider that pre-existent splenic diseases do not represent an absolute contraindication for NOM (HIV related splenomegaly). The splenomegaly induced by tropical diseases (especially malaria) require a conservative approach in the event of a trauma (NOM or splenorrhaphy). In Papua, New Guinea malaria is endemic with a high prevalence of ruptured pathological spleen but with a high preservation rate of over 70% (Waters). 

92% of all the patients with cirrhosis had an unsuccessfull NOM with 55% of fatal cases after surgery (splenectomy as a consequence of failed NOM) [**37**]. NOM failure is explained by altered spontaneous hemostasis associating with pre-existent portal hypertension syndrome (which leads to increased hydrostatic pressure within the parenchyma); there is also a clotting factor deficit in decompensated hepatic cirrhosis with a subsequent coagulopathy. Therefore, the mortality rate is directly correlated with increased PT values (prothrombin time), high lesion score and low serum albumin levels. Coagulopathy is a risk factor for a trauma patient with cirrhosis (Wahlstrom 2000; Tinkoff 1990; Morris 1990 - cit.37). It is imperative to operate to stop the bleeding if the patient has a pre-existent coagulopathy worsened by the ongoing hemorrhage. When preexistent coagulopathy is the one responsible for the bleeding following trauma, then the bleeding disorder should be tackled first and then decide whether or not surgical intervention is still required. Fang considers that cirrhosis is a contraindication for NOM.

Patients with a prolonged PT should not be approached by NOM in case of splenic trauma even if cirrhosis is not present [**38**].

**Religion**represents an important factor when treating splenic injury. Zieg and co. [**39**] presented the case of a type A hemophiliac patient, a Jehovah witness, with splenic trauma and favorable NOM outcome that was treated with recombinant factor VIII. There are 10 cases in English literature of hemophiliac patients and splenic trauma out of whom, 3 had an excellent outcome for NOM.

We now present the relative contraindications for NOM [**8,23,40**] which are basically criteria for a more cautious attitude when assessing and establishing the adequate treatment :

• multisystemic trauma;

• severe brain damage;

• another associated lesion interfering with the splenic lesion and possibly requiring surgical intervention ; in 1.7% splenic injury is associated with diaphragmatic lesion (Miller-41) and less than 1% of patients with blunt abdominal trauma exhibit hollow viscus injury (0.3% have intestinal perforation)[**42**].

• age>55 years [**43**];

• diseased spleen.

**The only absolute contraindication is represented by hemodynamic instability.**

The benefits of NOM [**26,44,45**] are:

• low morbidity and mortality; splenic preservation leads to lower early infections in adults;

• avoidance of a non-therapeutic laparotomy;

• no immediate/late complications that usually accompany a laparotomy;

• minimal blood transfusions

• decreased hospital stay (when other injuries prolonging the hospital stay coexist);

• maintened immunological function and prevention of OPSI. 

Potential drawbacks of NOM :

• overlooked injuries;

Allen and co (cit. 46) observed that 2.3% of NOM patients have had other associated injuries that were initially overlooked and required surgery later on (delayed diagnosis for over 6 hrs in 20% on patients with blunt abdominal trauma), but with many intra-abdominal complications. In Sartorelli’s study overlooked hollow viscus injuries totalized 0.8% of all cases [**23**].

• Impredictible time period for a second potential bleeding; the combination of increased use of NOM and decreasing hospital stays may increase the opportunity for outpatient rupture. 1.4 % of patients treated by nonoperative management required splenectomy and the median time to splenectomy was 8 days (Zarzaur- 47).

• Low splenic conservation rate following surgery after unsuccessfull NOM;

• A surgeon on call 24/7 and permanent clinical monitoring;

• Debates about the time period necessary for a complete recovery. 

Delayed surgical exploration could be increase the risk of hemorrhagic shock, major blood disorders, excessive blood transfusions and potential death. In 90% of cases the failure of NOM is evident in the first 50 hrs from the initial insult. Velmahos [**43**] identified 4 independent risk factors for an unsuccessfull NOM: splenic injury severity score, hemoperitoneum of over 300 ml, positive FAST, necessary blood transfusions. Statistically speaking, when all 4 factors are present, NOM will fail in 96% of cases.

Meyers [**26**], Uranus [**48**] and Wisner [**49**] pinpointed the following criteria for mandatory emergency surgery:

• persistent hemodynamic instability (despite aggressive fluid resuscitation);

• early recurrent hypotensive events (after adequate resuscitation);

• macroscopically positive diagnostic peritoneal lavage (in association with the previous criteria);

In Velmahos’s study [**11**] complications following NOM occurred in 40% of cases and consist of:

• persistent bleeding/ rebleeding;

This is obvious when an altered status is present along with occurrence/re-occurrence of internal bleeding signs, an increased number of transfused blood in order to maintain a normal systolic blood pressure, a worsening CT/US image and a significant drop in hematocrit and hemoglobin. In most cases persistent bleeding is the culprit; delayed bleeding occurs in 2- step splenic fractures (a real lesion- intrasplenic pseudoaneurysm) or in the case of a ruptured expanding subcapsular hematoma (water is moving through osmosis leading to increasing size of the hematoma).

• Post-traumatic splenic pseudocyst;

• Splenic abscess-rare; blood-spread infection or vecinity contamination are the main causes; the treatment consists of percutaneous drainage and in case of failure, splenectomy;

• Splenosis

• Postembolization asplenia (functional splenic failure);

• Pulmonary complications;

• Deep venous thrombosis;

• Blood transfusion-induced pathology(HIV, hepatitis C).

Schreiber (cit. 50) reckons that HIV infection risk, that of human leukemic virus with T cells and of hepatitis B and C from 1 unit of transfused blood is 1 in 34000 cases, 88% of them being hepatitis B and C.

**Unsuccessful NOM**

Occurs most frequently in the following circumstances:

• hemodynamic instability (systolic BP < 90 mmHg despite adequate resuscitation);

• age > 55 years old;

• > 4 units of transfused blood to maintain a hemoglobin level over > 10 g/dl;

• Persistent leucocytosis;

• The onset or aggravating sings of peritoneal irritation (suggesting further bleeding/ other overlooked injuries);

• Worsening imaging signs of splenic injury (repeated US exams)-post-traumatic splenic defect;

• Intra-abdominal compartment syndrome (intravesical pressure > 20 cm H2O).

According to Velmahos [**11**] the minimum time period necessary for a patient to be included in NOM protocol is 3 hrs.

The time interval between onset and reported NOM failure ranged between 6 and 94 hrs [**22**] with subsequent prolonged hospital stay (an average of 11.2 days). 67% of patients with unsuccessful NOM had contrast blush (hyperdense, well delineated, intraparenchymal contrast collection) [**40**]. Therefore, he concluded that the risk for failing NOT when contrast blush is present is 24-fold increased.

NOM failure can be explained by complications and by the constant pressure physicians find themselves to discharge patients as soon as possible; some failures are evident after discharge which means it is very important to identify any problem before that. Velmahos identified 2 independent risk factors for failing NOM: splenic injury ≥ 3 and more than 1 unit of transfused blood. When both factors are present NOM failing rate is as high as 97%; when none of these factors is present then NOM failing rate is 3% [**11**].

Unsuccessful NOM rate ranges between 2% and 31%.[**10,13,17,25,33,35,37,40,44,51-54**]. In Fang’s study [**37**] this rate was of 21.9% because 92% of his patients had liver cirrhosis.

Gavant’s and Federle’s retrospective studies (cit. 44) showed that contrast extravasation/ post-traumatic vascular injuries (contrast blush) visible on CT scans/ spiral CT scans with IV contrast are usually associated with an increased rate for unsuccessful NOM (these lesions may also be present in low grade injuries I, II).

**Fig. 1:**
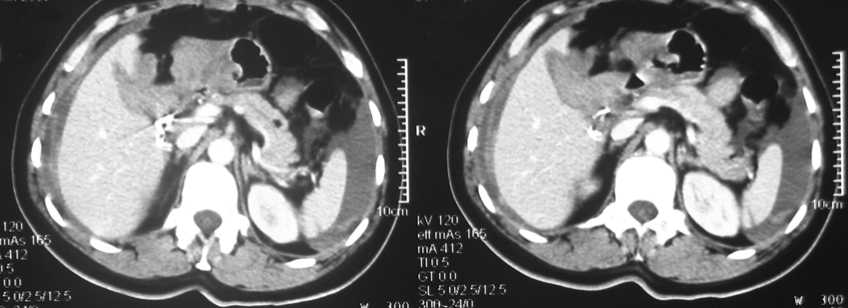
CT scan showing contrast extravasation (grade III splenic fracture); perisplenic and perihepatic hemoperitoneum.

**Fig. 2:**
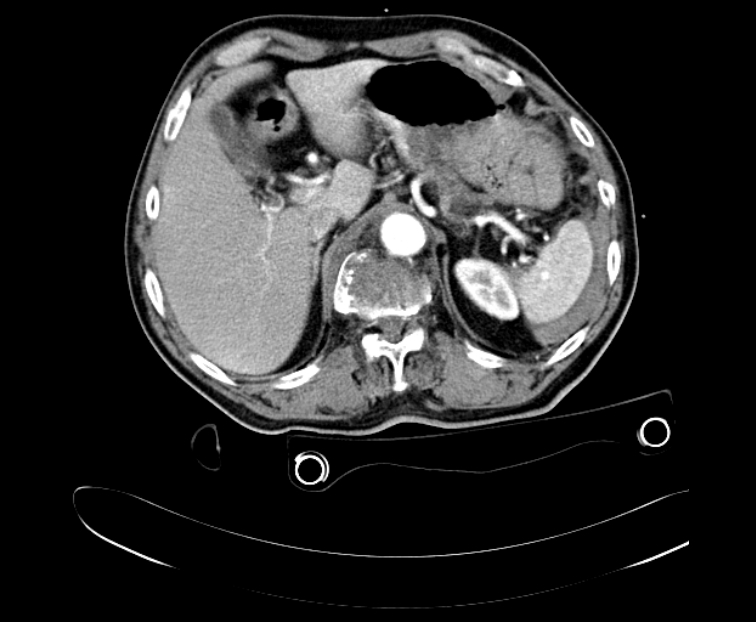
CT scan showing contrast blush in grade II splenic injury which was later confirmed by surgery; perisplenic hemoperitoneum.

Failing NOM in adults is equivalent with increased blood transfusions (with its risk) and impending surgery [**40**].

**Successful NOM**

In adults it ranges between 61.5% and 97%. [**10,33,36,55**]

Pachter [**36**] reports the following results: 53% in grade II injuries; 29% in grade III; 4% in grade IV; 1% in grade V lesions. The high percentage (97%) reported by Sclafani [**55**] is subsequent to the use of angiography and proximal angioembolization. NOM is successful in 97% of cases in children no matter the injury score (Velanovich cit. 8).

**Hospital stay**

It varies between 3 to 7 days when no other injuries are present to elicit a prolonged stay [**6,11,12,18,22**].

**Discharge recommendations** [**23,56**]:

• **Grade I-II lesions:**

o Avoidance of strenuous activities and sport (jogging, lifting >20 pounds, 1 pound=453.6 g),

o Avoidance of construction work for 6-8 weeks;

o Light activities (light work around the house, desk work, and light aerobic activity) 2 weeks after the initial injury.

o CT scan/US will be performed only if the clinical exam requires it.

• **Grade ≥III lesions **:

o Minimal activity for 1 week;

o Light activity 4-8 weeks;

o Avoidance of strenuous activities and sport for 10-12 weeks.

• **Grade IV, V lesions**:

o Avoidance of strenuous activities and sport for > 3 months.

o Mandatory CT scans or US.

**Splenic angiography (diagnostic and therapeutic)**

Recent NOM protocols for splenic trauma include angiography (diagnostic and therapeutic) as an efficient alternative [**57**]. Angiography can have a diagnostic purpose as well as therapeutic (vascular embolization and hemostasis).

The first angiographic embolization used Gelfoam (Katzen, 1976) and temporary balloon occlusion (Wholey, 1977) and were performed for hemostatic purposes prior splenectomy [**58**].

**Vascular lesions visible on angiography are** [**57,59,60**]:

• contrast extravasation inside or outside of spleen;

• vascular damage of terminal arteries (complete vascular transection);

• intraparenchymal arterio-venous fistula;

• intrasplenic pseudoaneurysm;

• vascular compression by subcapsular hematoma;

• variable degree of devascularization and irregularities in contrast filling (that includes Seurat spleen= small, spot-like, delineated/diffuse contrast collections).

**Indications for splenic angiography** [**61,62**]:

• grade 3, 4, 5 splenic injuries;

• vascular lesions visible on initial CT scan;

• active bleeding upon CT scan or contrast blush in a hemodinamically stable patient (upon repeated CT scans);

• unexplained decrease of hemoglobin level when no other lesions are present.

**Fig. 3:**
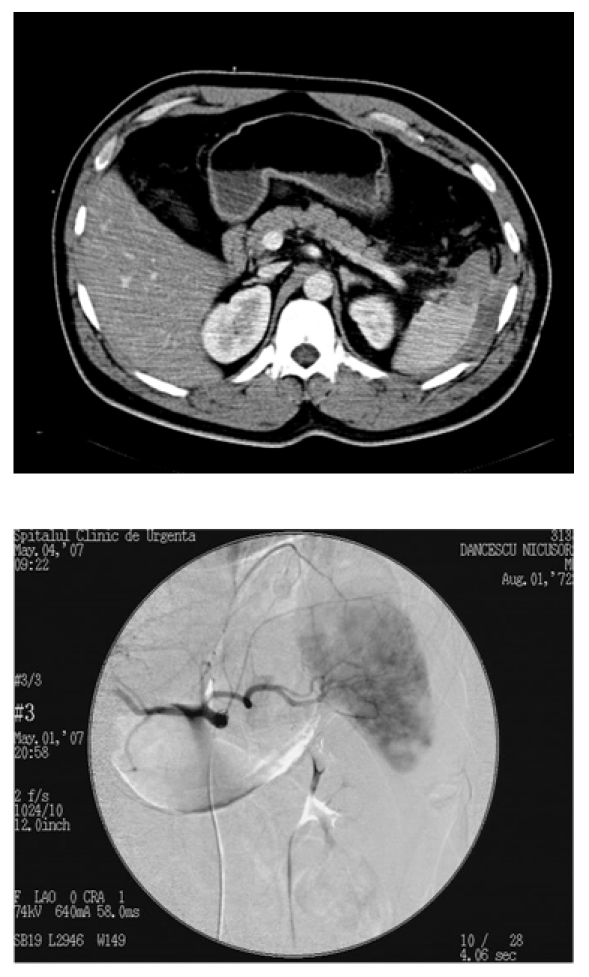
Grade III splenic fracture in a multiple trauma patient; splenic angiography does not show vascular damage- successfull NOM.

Splenic angioembolization (SAE) can be:

distal (supraselective)

• proximal (splenic artery)- achieved by using metal spirals (coils). It produces hemostasis by decreasing the blood flow and intrasplenic pressure; the viability of the remaining spleen is ensured by collateral blood flow (gastric arteries, omental arteries, pancreatic arteries). Sclafani [**55**] considers that the preservation of immunological functions is compatible with this procedure and even splenorrhaphy is facilited in case of surgical intervention.

• Combined.

Diagnostic and therapeutic (embolization) angiography is performed after CT scans showed intrasplenic vascular damage. Embolization is carried out only if there is angiographic confirmation of the lesion [**61**].

Second-look angiography is useful in recurrent bleeding and after an initially negative angiography (10%) (63 Haan). Haan employed preferentially distal SAE for small grade lesions and combined SAE for severe injuries (however with almost no statistical difference). Haan [**63**] also believes that “delayed vascular emergencies” (term first introduced by the Memphis group) are basically delayed diagnoses that become evident when performing angiography for severe splenic injuries (grade 3, 4, 5). The Memphis group (Davis, Fabian, Croce) proved that initial CT and angiographic scans can skip vascular injury due to arterial spasm at the moment of the examination but can later become clinically detectable; spiral CT scans identified 80% of all vascular lesions that were initially unnoticeable (spiral CT is used as a screening test for angiography). The only statistically significant failure risk for NOM is the arterio-venous fistula which is treated not only by proximal SAE but by a more direct approach-distal SAE [**64**]. 

The conclusions inferred by Haan’s study are [**65**]:

• Proximal SAE is a much more useful therapeutic method than distal embolization (because it decreases the splenic perfusion pressure); the exception is an arterio-venous fistula;

• The immunological consequences of proximal embolization are still unclear and require further investigation;

• The use of SAE decreases by 20% the failure rate of NOM in grade 4 and 5 injuries;

• SAE proved to be superior to surgical intervention when dealing with blunt splenic trauma in multiple trauma patients with brain injury.

SAE is a useful and efficient method for NOM but it is necessary in only 7% of cases [**61**].

**SAE indications** [**64,66**]:

• **Proximal SAE**: it is indicated in hilar lesions;

o >3 distinct peripheral vascular lesions;

o the injury affect more than 50% of the splenic parenchyma.

• **Selective SAE**: limited vascular injuries. It is proficient because it allows proper hemostasis and adequate perfusion to remaining organ.

• **Combined SAE**: for multiple vascular injuries (high injury scores).

It is recommended to perform multiple CT scans after SAE in order to monitor the vascular damage, pseudoaneurysm formation, size of infarcted area and existence of localized infection (splenic abscess).

SAE represents an elegant alternative and is now part of all NOM protocols in trauma centers.

**SAE induced complications** [**59,63,67-71**]

o Major complications (19%-28.5 %)

o Bleeding- it is the most common complication caused by delayed diagnosis of pseudoaneurysms and late pseudoaneurysm formation;

o Overlooked injuries: usually diaphragmatic, pancreatic;

o Infection- splenic abcess, sepsis;

o Splenic atrophy;

o Iatrogenic arterial damage;

o Acute renal failure after contrast administration

o Deep venous thrombosis.

o Minor complications (23%-61.9 %)

o Splenic infarction: in 27% of cases after distal SAE and in 20% of cases after proximal SAE. Most of them are asymptomatic but it is believed that a splenic infarction is significant when a devascularization of >25% of splenic parenchyma occurs (upon repeated CT scans);

o Migration of embolic material: spiral that migrates in proximal SAE needs extraction.

o Angiographic vascular dissection: it is usually asymptomatic and non-occlusive (femoral artery, splenic artery).

o Vascular damage when inserting the catheter( arterio-venous fistula)

o Persistent pain at the catheter insertion site

o Hematoma on the puncture site. 

o Post-emobolization syndrome- includes symptoms such as general discomfort, fever, local pain and/or leucocytosis which generally persist for 3-5 days; if blood cultures are negative and no signs of infection are present then it is considered to be a rather benign complication. It is self-limiting and it is caused by extensive tissue necrosis or intravascular thrombosis subsequent to a successful embolization.

o Pleural and pulmonary complications;

o Thrombocytosis;

o Allergic reactions to contrast

In Shih series [**71**] 28.5 % of patients had major complications including 4 cases of postprocedural bleeding that might be attributed to the use of Gelfoam as an embolizing agent. 

**CT findings after SAE** [**72**]

Areas of spleen infarction appear after SAE that have certain characteristics:

• Infarction appeared in 63% of cases after proximal SAE, but only in 20% of cases the area extended over more than 50% of splenic parenchyma. These areas are usually small in size, multiple, situated at the splenic border and heal completely.

• Infarction areas after distal SAE occur in 100% of cases with only 9% of cases affecting over 50% of the splenic parenchyma. They are usually a unique, large area immediately beneath the embolized blood vessel and heal completely in most cases.

Statistically speaking distal SAE triggered more splenic infarctions than proximal SAE.

• Combined SAE trigger splenic infarction in 71% of cases; in 20% of them more than 50% of splenic parenchyma was affected.

When air bubbles are visible within the splenic parenchyma it is necessary to rule out a splenic abscess. Likewise, the presence of air-fluid level in a subcapsular collection suggests the development of a splenic abscess (which can be drained percutaneously).

The immune alteration after SAE remains unclear. In a recent study Shih et al. [**71**] showed that SAE dysregulates the nuclear factor (NF)-kB translocations and aggravates the cytokine response in patients with spleen injury. Nakae [**73**], in a recent study finds that splenic preservation (embolization, splenorrhaphy, partial splenectomy) not have advantages over splenectomy in immunologic indices including levels of IgM and 14 serotypes of anti-Streptococcus Pneumoniae antibodies. Tominaga’s results [**74**] suggest that the immunologic profile of embolized patients is similar to controls. He tested IgM, IgG, C 3 complement, complement factor B, CD3, CD4, CD8 (helper and suppressor T-cells), complete blood counts and HIV status and found that splenic immunocompetence is preserved at a minimum 3 months after embolization. Consequently the immunization may not be necessary. However, larger studies are useful to make definitive vaccination recommendations.

NOM represents an effective and safe alternative for selected patients with splenic trauma [**75,76**]. When dealing with splenic trauma NOM is the rule and not the exception [**9**] with its success relying upon adequate clinical assessment.

The utilization of mobile digital subtraction angiography directly into the trauma resuscitation area shortened the time required to restore normal physiology (more rapid reversal of acidosis, coagulopathy and hypothermia- “triad of death”- due to shortening the time required for hemostasis) (Morozumi-77).

Recent studies suggests that early surgical intervention should be considered in blunt splenic injured patients with contrast extravasation and ISS ≥ 25 (Fu-78); Velmahos [**79**] identified 2 independent predictors of NOM failure: grade V blunt splenic injuries and the presence of a brain injury. Jeremitsky [**80**] has evaluated the role of splenic embolization as an adjunct for NOM and found that it increased splenic preservation success. In his opinion the markers of greater injury severity are associated with an increased risk for NOM failure and substance abuse represents an independent predictor of NOM failure. Splenic angioembolization represents a valid and effective option in patients with severe splenic injuries and/or active bleeding (81,Franco-82).

The use of splenic angioembolization for traumatic injuries was initiated at our institution in 2009. The first successful splenic angioembolization in trauma in Romania was performed at Emergency Hospital Bucharest and published in “Chirurgia” in 2010 (Venter-83).

As a conclusion: “ in a hemodinamically stable patient, with a major splenic injury, proximal SAE has the same effectiveness as splenectomy but with a low number of units of transfused blood and a low mortality rate” -Salvatore Sclafani.

**Acknowledgement**. Our entire gratitude to Dr. Ioana-Iftimie Nastase for helping us translate this manuscript.
